# Comparison of Next-Generation Sequencing, Real-Time PCR and HRM-PCR for *Helicobacter pylori* Detection in Pediatric Biopsies

**DOI:** 10.3390/microorganisms13102344

**Published:** 2025-10-13

**Authors:** Tomasz Bogiel, Anna Szaflarska-Popławska, Dariusz Grzanka, Marcin Woźniak, Tomasz Gosiewski, Agnieszka Krawczyk

**Affiliations:** 1 Department of Propaedeutics of Medicine and Infection Prevention, Ludwik Rydygier Collegium Medicum, Bydgoszcz Nicolaus Copernicus University in Toruń, 9 Maria Skłodowska-Curie Street, 85-094 Bydgoszcz, Poland; 2 Department of Pediatric Endoscopy and Gastrointestinal Function Testing, Ludwik Rydygier Collegium Medicum in Bydgoszcz, Nicolaus Copernicus University, 85-094 Bydgoszcz, Poland; anna.szaflarska@cm.umk.pl; 3 Department of Clinical Pathomorphology, Ludwik Rydygier Collegium Medicum in Bydgoszcz, Nicolaus Copernicus University, 85-094 Bydgoszcz, Poland; d_grzanka@cm.umk.pl; 4 Department of Forensic Medicine, Ludwik Rydygier Collegium Medicum in Bydgoszcz, Nicolaus Copernicus University, 85-094 Bydgoszcz, Poland; marcinw@cm.umk.pl; 5 Department of Molecular Medical Microbiology, Chair of Microbiology, Jagiellonian University Medical College in Cracow, 31-121 Kraków, Poland; tomasz.gosiewski@uj.edu.pl

**Keywords:** *Helicobacter pylori* diagnostics, high resolution melting, HRM, next-generation sequencing, PCR-HRM, real-time PCR

## Abstract

In 40 unique pediatric biopsy samples, this study aimed to compare the obtained results of *Helicobacter pylori* DNA detection using next-generation sequencing (NGS), a real-time PCR-based IVD-certified kit and an established high resolution melting real-time PCR-based method for *H. pylori*-specific *ureA* gene. From the same group, the *H. pylori* DNA was identified in 16 (40.0%) samples in both real-time PCR-based methods, with quantification cycle (Cq) values ranging from 17.51 to 32.21 for the IVD kit. NGS was able to detect *H. pylori* DNA in 14 (35.0%) samples, with read counts between 7768 and 42,924. While all three methods showed similar detection rates, both PCR variants were slightly more sensitive, identifying *H. pylori* in two additional samples not detected by NGS. The study highlights the strengths and limitations of each method. NGS, though promising due to its high sensitivity and ability to detect low bacterial load, is still limited by its cost and complexity. Despite these challenges, NGS could complement PCR in diagnosing difficult or ambiguous cases, enabling the detection of multiple pathogens simultaneously. Especially when other infectious etiologies are suspected, NGS could be considered, though PCR variants remain a more attractive and cost-effective option for routine *H. pylori* detection.

## 1. Introduction

*Helicobacter pylori* (*H. pylori*) is a Gram-negative, microaerophilic bacterium, usually spiral-shaped; however, it can also appear as a rod or coccoid shape [1]. The presence of the flagellum enables *H. pylori*’s movement. Importantly, *H. pylori* is able to form biofilms on the gastric mucosa. Biofilm formation enhances bacterial survival by providing a protective habitat against both the host immune response and antimicrobial agents. This ability is increasingly recognized as a critical factor in the chronicity of infection and the emergence of antibiotic-resistant strains, making eradication therapy more challenging [2]. Moreover, *H. pylori* can survive in the acidic gastric environment due to the production of urease, which catalyzes the hydrolysis of urea into ammonia, thereby neutralizing the low pH of gastric juice. Infections caused by *H. pylori* are strongly related to many gastroduodenal disorders [3]. Upper gastrointestinal infections with *H. pylori* are common and their prevalence ranges between 9 and 80%, depending on region and population [3,4].

A bacterial culture of biopsy samples has been accepted as the gold standard method in diagnosing *H. pylori* infection [5], which presents 100% specificity [6]. Nevertheless, the sensitivity of this method is low and varies, and also depends on many factors, including the conditions of sample collection, storage or transport, time interval between sampling and sample plating for culture purposes, occurrence of commensal microbiota in clinical specimens, experience of the laboratory staff, quality of the sample taken, etc. [6,7]. Moreover, a reliable diagnosis using bacterial culture requires discontinuing proton pump inhibitors for at least 2 weeks and antibiotics for at least 4 weeks preceding endoscopy [8]. Otherwise, a false-negative result may be obtained. In turn, rapid urease tests may be negatively influenced by the presence of other urease-producing microorganisms in the stomach, e.g., *Staphylococcus urealyticum*, which can lead to false-positive test results [9].

**Figure 1:**
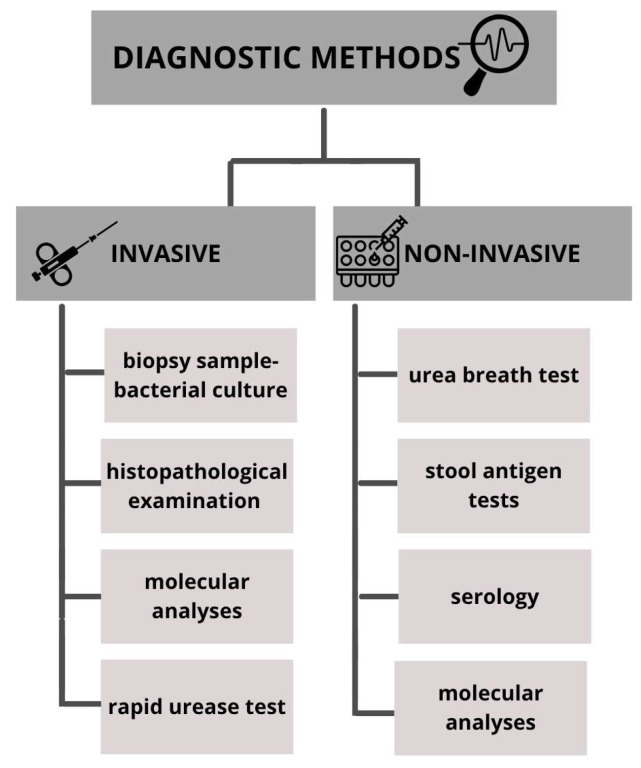
Methods used in the diagnosis of *Helicobacter pylori* infections.

Numerous methods are described for detecting the presence of *H. pylori*; each diagnostic approach has its own advantages, disadvantages and limitations. In general, diagnostic methods can be divided into invasive and non-invasive (Figure 1). Invasive methods require taking a biopsy sample during endoscopic examination and are based on gastroscopy images, histopathological examination of tissue samples, rapid urease test, culture and molecular analyses. Non-invasive methods include stool antigen tests, serological investigation of *H. pylori*-specific antibodies and urea breath test [5,10].

Since the development of molecular biology methods and their implementation in routine microbiological diagnostics, polymerase chain reaction (PCR) has increasingly been used to detect the presence of DNA of several microorganisms from different clinical specimens, including *H. pylori* from stool, saliva, tissue samples or gastric juice. PCR is characterized by high sensitivity and specificity, greater than 95% in comparison to other conventional methods. Additionally, PCR enables the detection of any mutations leading to antibiotic resistance, which significantly speeds up diagnosis and improves the treatment process [7].

The PCR method modification called high resolution melting (HRM) has gained popularity in microbiological diagnostics over the past several years [11]. It is a fast and sensitive PCR-based technique that enables the simultaneous detection of genetic sequences as well as differences between them, such as mutations, polymorphisms or structural changes in the tested material [12,13,14]. HRM is based on the analysis of the amplicon melting curves and the melting temperature (Tm) of the amplified DNA fragments. During this process, the sample is gradually heated, and changes in fluorescence dyes’ incorporation related to DNA sequences and resulting denaturation are recorded. Due to the high resolution of the fluorescence measurements, this method can also detect minor differences in DNA composition, such as small changes in nucleotides sequences. HRM allows for a rapid and precise detection of pathogens based on their unique genetic sequences. It can be used not only to identify microorganisms in clinical samples, but also, even more importantly, to detect mutations that contribute to drug resistance [12,14,15,16].

Another molecular method that is gaining more and more supporters and is being used in microbiology on an increasingly large scale is next-generation sequencing (NGS). While PCR is increasingly used for the detection of *H. pylori* [6,8,17,18,19], the possibility of implementing NGS has only been described by a few researchers and these studies mainly focus on investigating a wide range of basic mechanisms in *H. pylori*, such as the presence of genes associated with bacterial virulence or antibiotic resistance [20,21]; biofilm formation [22]; the characterization of methylases, hydrolases, exo- and endo-ribonucleases [23,24,25] or the transcriptional response of *H. pylori* to exposure to different environmental conditions such as salt concentrations, pH conditions or chemical agents [26,27,28,29]. However, there is a lack of research on the potential use of NGS in standard diagnostics of *H. pylori* infections, the usefulness of the method in determining infection in complex samples/cases, and comparison of the effectiveness of NGS with other diagnostic methods. Traditional Sanger sequencing, developed in the late 1970s, has long been considered the gold standard for DNA sequencing due to its high accuracy and reliability in determining nucleotide sequences [30]. However, this method is limited by relatively low throughput, high cost per base and the need for prior amplification of the target region, which makes it less suitable for large-scale or complex microbial community analyses [31]. In contrast, NGS enables massively parallel sequencing of millions of DNA fragments simultaneously, providing much greater depth of analysis and the ability to detect mixed or low-abundance bacterial populations in clinical samples [32,33]. While Sanger sequencing remains valuable for targeted gene analysis and validation of genetic variants, NGS offers superior scalability and sensitivity, making it more promising for comprehensive pathogen detection and characterization in a diagnostic context.

Therefore, this study aimed to compare the results obtained with IVD-certified real-time PCR, NGS method and “in house” PCR-HRM methodology (for inconclusive results, as a decisive method) for detecting the presence of *H. pylori* DNA in a unique batch of 40 pediatric biopsy samples.

## 2. Materials and Methods

### 2.1. Patients and Biopsy Samples

The research materials were gastric biopsies collected between December 2018 and August 2019 from pediatric patients aged 3–17 years (*n* = 40). Patients were examined at the Department of Pediatric Endoscopy and Gastrointestinal Function Testing of Ludwik Rydygier Collegium Medicum in Bydgoszcz, Nicolaus Copernicus University in Toruń, Poland. The inclusion criteria were as follows: patients included in the study were between 3 and 17 years old, initial diagnostics of chronic or recurrent non-functional dyspeptic symptoms, and written parental (for children < 16 years) or both parental and patient consent to upper gastrointestinal endoscopy. The study protocol was approved by the Institutional Review Board of Ludwik Rydygier Collegium Medicum in Bydgoszcz Nicolaus Copernicus University in Torun; Ethics Committee approval code: KB 772/2018, approved on 20 November 2018.

### 2.2. Methods

Immediately after the collection steps, tissue samples were preserved in a transport medium (BD BBL™ Port-A-Cul™ transport systems, Becton Dickinson, Franklin Lakes, NJ, USA) and delivered to the Microbiology Department, Ludwik Rydygier Collegium Medicum in Bydgoszcz, Nicolaus Copernicus University, Bydgoszcz, Poland, where the DNA was isolated, and all the further analyses were carried out without unnecessary delays.

#### 2.2.1. DNA Isolation from the Biopsy Samples

The tissue samples were subjected to mechanical lysis for 1 min using a manual homogenizer (Squisher-Single, ZymoResearch, Irvine, CA, USA). Then, the samples were subjected to 30 min digestion in 200 microliters of trypsin solution (5 mg/mL, Trypsin EDTA solution, Sigma, Darmstadt, Germany) at 37 °C in order to increase DNA isolation efficiency. Subsequent steps of DNA extraction were performed using the GeneProof PathogenFree DNA Isolation Kit (GeneProof, Brno, Czech Republic) according to the manufacturer’s instructions. The isolated samples containing DNA were frozen at −20 °C until further investigation.

#### 2.2.2. Real-Time PCR Using the AmpliSens^®^ *Helicobacter pylori*-FRT PCR Kit

The samples obtained through the isolation process were tested for the presence of *H. pylori* DNA. Detection was conducted using the AmpliSens^®^ *Helicobacter pylori*-FRT PCR Kit (AmpliSens, Bratislava, Slovakia) based on real-time PCR for *16S rRNA* gene. According to the manufacturer’s documentation, the assay amplifies a specific region of the *H. pylori 16S* rRNA gene; however, the exact amplicon length and primer sequences are proprietary and not publicly available. After preparing the reaction mixture, the samples were added to multi-well plates (Roche, Basel, Switzerland) and transferred to a Cobas z480 PCR analyzer (Roche, Basel, Switzerland). All the samples were tested at least in duplicate to check the reproducibility of the PCR results. The real-time PCR results were interpreted based on the quantification cycle (Cq) threshold values, as medium values of the subsequent investigations.

Each round of investigations was supplemented with negative control containing molecular biology-grade water (EurX, Gdańsk, Poland) instead of DNA. The DNA extracted from the reference strain (*H. pylori* DSMZ 21031) was used as a positive control in each round.

#### 2.2.3. Next-Generation Sequencing

Sequencing of the bacterial 16S rRNA-encoding V3 and V4 regions was carried out using the NGS method, through sequencing by synthesis. The primer sequences were the following: F: CCTACGGGNGGCWGCAG and R: GACTACHVGGGTATCTAATCC.

The overhang adapter sequences for MiSeq sequencer [34] (Illumina, San Diego, CA, USA) were added to the internal primer attached to the 5′ end, i.e.,: F: TCGTCGGCAGCGTCAGATGTGTATAAGAGACAG and R: GTCTCGTGGGCTCGGAGATGTGTATAAGAGACAG. This primer pair amplifies a fragment of approximately 550 bp covering the V3-V4 hypervariable regions of the bacterial *16S* rRNA gene.

The composition of the reaction mixture included 1 μL of DNA, 12.5 μL of Kapa Biosystems reagent (Roche, Basel, Switzerland), 0.5 μL of each primer (10 mmol/L, Genomed, Warsaw, Poland), 10.5 μL of molecular biology-grade water (EurX, Gdańsk, Poland). Amplification conditions included denaturation at 95 °C for 3 min and then 30 cycles of the following conditions: 95 °C—30 s, 55 °C—30 s, 72 °C—30 s. Subsequent steps in genomic library preparation (purification, indexing, quantification, pooling) were prepared according to the Illumina protocols with minor modifications as previously described [34,35,36].

#### 2.2.4. PCR-HRM for *H. pylori ureA* Gene Detection (Verification Method)

Additional research was performed to verify the presence of *H. pylori* DNA in 40 investigated samples (see Table 1) by “in-house” real-time PCR for the *ureA* gene detection. High resolution melting curve genotyping analysis was additionally applied for this purpose. The mentioned methodology was applied once for the samples with concordant results and twice for 16 samples with discordant results between the real-time PCR-based diagnostic kit and NGS results. The primers used for the *ureA* gene amplification were as follows: F: AGTTCCTGGTGAGTTGTTCTT and R: TGGAAGTGTGAGCCGATTT, according to the study by Hasyanee Binmaeil et al. [16]. This primer set amplifies a 139 bp fragment of the *H. pylori ureA* gene.

CFX Opus Dx Real-Time PCR Detection System (BioRad, Feldkirchen, Germany) was applied for the purpose of the *ureA* gene fragment amplification and HRM curve preparation and, therefore, confirmation of the presence of *H. pylori*.

DNA extracted from a reference strain of *H. pylori* (DSMZ 21031) was used as a positive control, while molecular biology-grade sterile water (EurX, Poland) was applied as a negative amplification control. Positive amplification peaks were determined to be within the temperature range of the reference strain gene (81–82 °C). Positive and negative controls were used to evaluate reliability in each batch of the research round.

The amplification reactions were also performed with the application of molecular biology-grade sterile water (EurX, Gdańsk, Poland), primers (Genomed, Poznań, Poland) and the 5x HOT FIREPol^®^ EvaGreen^®^ HRM Mix (no ROX) reaction mixture (Solis BioDyne, Tartu, Estonia). The reaction volume for one sample consisted of 20 µL, with the following ingredients: 4 µL of HRM Mix, both primers at the final concentration of 0.2 µM, water (5 µL) and 1 µL of DNA template. The amplification program consisted of the following: initial denaturation at 95 °C for 12 min, then 50 cycles of amplification, each consisting of denaturation at 95 °C for 10 s, primer annealing at 60 °C for 20 s and primer extension at 72 °C for 20 s.

For the confirmation of the real-time PCR products’ specificity, PCR-HRM was performed to detect the actual *H. pylori* species-specific *ureA* gene amplification for all the tested samples subsequently after real-time PCR and NGS evaluation (*n* = 40, altogether). The melting curve of the amplified product (high resolution melting analysis) was investigated, using the following amplicon melting curve analysis protocol: 95 °C for 5 s, 65 °C for 60 s and constant heating until reaching 97 °C (with ramp rate 0.11 °C/s and five read-outs per °C) using constant fluorescence readings. The amplification conditions mentioned above were set according to the PCR-HRM EvaGreen Mix manufacturer’s instructions (SolisBiodyne, Tartu, Estonia).

#### 2.2.5. Bioinformatics/Statistical Analysis

Sequencing quality was assessed initially using FASTQ files obtained and FastQC v.0.11.9. Metagenomic sequencing data were analyzed using 16S Metagenomics v 1.1.3 pipeline, available on Illumina’s BaseSpace server. Both available reference databases (RefSeq RDP 16S v3 May 2018 DADA2 32 bp and Greenegenes May 2013 32 bp) were used for species identification and counting. The results obtained from those analyses were exported and processed using Microsoft Excel 365 for data organization and visualization. Statistical analyses (calculation of means, standard deviations and assessment of reproducibility) were performed using Statistica v13.3 (TIBCO Software Inc., Palo Alto, CA, USA).

## 3. Results

The real-time PCR results were interpreted based on the quantification cycle (Cq) threshold values, while PCR-HRM results were interpreted based on the melting curve shape analysis and melting temperature for the amplicons. *H. pylori* DNA was detected in 16 (40.0%) biopsy samples by real-time PCR (Table 2). The Cq values for positive samples ranged from 17.51 to 32.21 (mean ± SD reached 22.43 ± 3.3) and are presented in Table 2.

The NGS method showed the presence of *H. pylori* DNA in 14 (35.0%) samples (Table 2). The results were interpreted based on the number of reads, reaching 7768 to 42.924 non-chimeric read outs (Table 1).

The results of PCR-HRM, considered conclusive in case of discrepant results, were positive for 16 samples, the same as for real-time PCR (for examples see Supplementary Material Figures S1–S3.

During our study, sequence analysis of the amplified regions revealed a high specificity for *H. pylori*. The *ureA* gene primers did not show significant homology with other gastric bacteria, minimizing the risk of false-positive results. For the *16S* rRNA V3-V4 regions targeted in NGS, some sequences shared moderate similarity with other *Helicobacter* species or closely related gut microbiota. However, the combination of read depth, quality filtering and taxonomic classification allowed unambiguous identification of *H. pylori* in all samples. No sequences were observed that could significantly confuse the analysis or lead to misidentification.

To ensure the specificity of molecular biology-based results, all amplified products were verified using the suitable approach. For real-time PCR, all samples were tested at least in duplicate to ensure reproducibility. Each PCR run included a negative control (molecular biology-grade water) and a positive control *(H. pylori* reference strain). The specificity of HRM-PCR products was confirmed by the characteristic melting temperature of *H. pylori* amplicons, which allowed unambiguous discrimination from non-specific products. In addition, NGS sequencing of the *16S* rRNA V3-V4 regions provided nucleotide-level confirmation, allowing precise identification of *H. pylori* in all samples and ensuring that the detected sequences were correctly assigned to the target organism.

## 4. Discussion

Currently, *H. pylori* infection can be detected by numerous methods, among others: histological examination, stool antigen testing, bacterial culture, serological tests, urease testing or molecular investigation. The choice of diagnostic method is influenced by many factors, such as test sensitivity and specificity, clinical specimen type, available hardware facilities, clinical circumstances or cost-effectiveness of the testing strategy. In medical practice, a rapid, accurate and cost-effective method for detecting the presence of pathogenic microorganisms, including *H. pylori*, is desirable.

The PCR method has been used in routine microbiological diagnosis of many infections for many years. There are many studies that also include this method in the diagnostics of *H. pylori* infection. In our previous studies, we have shown that the results obtained by PCR provide reliable results, consistent with histopathological examination [6]. Additionally, Lo et al. [37] showed that the sensitivity of PCR was significantly higher than that of rapid urease tests, histology and culture (respectively 91% vs. 66%, 43% and 37%). In addition, the PCR method was characterized by greater specificity than serological investigation (100% vs. 65%). In the study conducted by Noh et al. [38], the real-time PCR method showed a positive result rate of 97.1% while the rapid urease test detected the presence of bacteria in only 82.9% of samples. Moreover, thanks to the real-time PCR method, it was possible to simultaneously detect clarithromycin resistance, which shows another advantage of molecular methods over conventional ones. This will be the goal of our next study, as we were capable of collecting numerous samples of these unique pediatric specimens.

In turn, Trung et al. showed that the percentage of positive results obtained using the multiplex PCR was 72.1%, whereas using the rapid urease test it was only 38.3%. It is worth adding that the accuracy rate of the urease test was 85.07% while the PCR showed an accuracy of 83.08% [39]. These results indicate that both methods have high accuracy for the diagnosis of *H. pylori*, but rapid urease test should always be used in combination with molecular methods to reduce the rate of false-negative results.

NGS is a relatively new method, used mainly in commercial studies or microbiome analyses. It is rarely used in standard microbiological diagnostics, mainly due to costs, the need for specialized equipment and bioinformatic analysis of the obtained results. Nevertheless, it is increasingly being implemented in diagnostics, as a supplement or confirmation of commonly used methods. Unlike traditional sequencing methods such as Sanger sequencing, NGS enables the sequencing of millions of fragments simultaneously, offering unprecedented speed, throughput and accuracy. In our study, we checked whether the NGS method is useful in diagnosing *H. pylori* infections and if its effectiveness is comparable to another, more commonly used molecular biology method, an IVD-certified real-time PCR kit. We showed that the percentage of positive samples was similar using both methods; however, the PCR method was slightly more effective. Moreover, its results were confirmed using PCR-HRM, both detecting *H. pylori* DNA in two additional samples in which NGS did not detect the genetic material of this bacterium.

PCR-HRM seems to be an effective method for confirming the presence of *H. pylori* DNA (either infection or colonization). In our study, *H. pylori* DNA was detected using the HMR method based on the presence of the *ureA* gene. The only similar study comes from Nigeria (Lagos) and also includes 40 biopsy samples, comprising 20 biopsies obtained from the antrum and 20 from the corpus of 20 patients undergoing endoscopy for duodenal ulcer investigation. However, the study does not compare this method with any other molecular tests, making it impossible to determine its concordance with any other methodology. Nevertheless, the researchers indicate that the applied HRM demonstrated high efficiency in detecting small amounts of *H. pylori* DNA, and the correlation coefficient and amplification efficiency were found to be promising for both *ureA* (0.994 and 90.35%, respectively) and *16S rRNA* (0.997 and 97.94%, respectively) [40]. Also, Noh et al. used HRM to identify the presence of *H. pylori*, but based on the *23S rRNA* gene. Among the 70 analyzed samples, PCR-HRM detected *H. pylori* in 97.1% of cases, while the rapid urease test showed a positivity rate of only 82.9%. Moreover, among these samples, resistance to clarithromycin, determined by minimum inhibitory concentration, was confirmed in 18.6% (13/70) of samples. Additionally, fluorescence melting curve analysis revealed that 84.6% (11/13) of these resistant cases carried point mutations in the *23S rRNA* gene. The PCR-HRM assay proved to be more effective than rapid urease test in detecting *H. pylori* infection and demonstrated strong reliability in confirming clarithromycin resistance compared to antimicrobial susceptibility investigated in cultures [38].

In the context of *H. pylori* infections, the HRM method is used not only for detecting the bacterium but has also been described in many other applications. Silva-Fernandes et al. have shown that HRM can play an important role in analyzing DNA polymorphisms in genes encoding DNA repair enzymes in the progression of cancers associated with *H. pylori* infection [41]. In turn, Xie et al. used the HRM method to assess the methylation status of the DLEC1 and PBX3 promoters in peripheral blood, which are associated with the risk and prognosis of gastric cancer [42]. A similar application of the HRM method in relation to research on carcinogenesis and the prevention of gastric cancer has been described by Iranian [43], Japanese [44] and Chinese [45,46] research teams.

An interesting aspect is the use of HRM to identify polymorphisms in *H. pylori* strains that are resistant to clarithromycin in the analysis of the microbiome composition isolated from cases of gastric inflammation [43]. The method showed a good performance (81.58% accuracy and 100% specificity) in the Shenzhen validation cohort. The results were fully consistent with whole-genome sequencing, showing a complete match for the A and G alleles in the *23S rRNA* gene at position 2143, and it was calculated that the detection limit can be as low as 0.005 ng/μL. Moreover, HRM effectively identified wild-type and mutant *H. pylori* directly in samples, showcasing its applicability for culture-free resistance testing. This approach significantly simplifies the determination of *H. pylori* antibiotic resistance, offering a more effective alternative to traditional techniques [47].

Similarly, Beheshtirouy et al. indicate that the HRM method can be used to assess drug resistance. The researchers, based on paraffin-embedded tissue sections from gastric cancer, in which the presence of *H. pylori* was confirmed, examined mutations in the *23S rRNA* gene of *H. pylori*, which are associated with clarithromycin resistance, as well as in the *rdxA* and *frxA* genes of the bacterium, which may be linked to metronidazole resistance [48]. A similar application of the HRM method to study drug resistance is demonstrated in the research conducted by Nahm et al. The researchers aimed to identify the A2142G/A2143G mutations in the *23S rRNA* gene, associated with clarithromycin resistance, and achieved over 90% concordance of the HRM with traditional methods [15].

Several research groups have also previously highlighted usefulness of PCR-HRM in microbiological diagnostics of gastrointestinal tract, not only in the context of *H. pylori*. Ngui et al. used this method to identify and differentiate five human hookworms. The researchers noted that the developed HRM assay is a rapid, effective and simple technique for identifying and distinguishing hookworm species. Importantly, it does not require multiplexing, DNA sequencing or post-PCR processing. It is also worth mentioning the high effectiveness of identification using this method. The HRM method demonstrated the same effectiveness as microscopy, showing a positive result in 58 of the examined samples, while nested PCR showed a positive result in only 47 probes [49]. Lamien-Meda et al. also demonstrated the high efficacy of HRM in diagnosing parasitic diseases, focusing on gastrointestinal parasites. The researchers achieved 95.8% concordance with conventional methods, indicating high sensitivity and specificity of the real-time PCR protocol, based on high resolution melting [50].

In turn, in a study conducted by Kafi et al., multiplex high resolution melting assay was used for the simultaneous detection and differentiation of five major bacterial pathogens responsible for urinary tract infections (UTIs) directly from urine samples. Compared to culture, the specificity of the HRM assay ranged from 99.3 to 100%, with a sensitivity of 100% for all the tested pathogens. This method has been shown to be both specific and sensitive, providing results in less than 5 h, which represents a significant improvement over traditional culture methods that usually require 24–48 h [51].

There are currently no studies comparing NGS- and PCR-based methods in the diagnosis of *H. pylori*, so we cannot relate our results to those obtained in other research that includes this specific bacterium. However, there are many studies comparing these two methods, among others, in the diagnosis of urinary tract infections, and their results are consistent with ours, showing a slight advantage of PCR over NGS. Zhao et al. [52] showed that PCR had a sensitivity of 99% and a specificity of 94% for detecting UTI, while NGS had a sensitivity of 90% and a specificity of 86%. Nevertheless, the researchers concluded that both PCR and NGS have great potential for routine diagnosis of UTI and have predominance over culture-based methods. There are interesting studies comparing NGS and PCR in the diagnosis of HPV in plasma samples and oral rinsing fluids. Researchers have shown that HPV DNA detection by NGS had 70% sensitivity compared to 20.6% sensitivity by qPCR (*p* < 0.001). In oral rinse, NGS also demonstrated higher sensitivity (75.0%) compared to qPCR (2.1%, *p* < 0.001) [53]. This study shows limitations of PCR in the analysis of some materials; therefore, in case of doubtful or unclear results, it is good to verify the result with another sensitive method, such as NGS.

Several other studies have also speculated about the possibility of implementing NGS for routine diagnostic schemes, demonstrating the many advantages of this method [54,55,56]. An important advantage of the NGS method is the detection of even very low abundance of bacteria. Thorell et al. [57] showed that meta-transcriptomics study using NGS allows for detection of actively replicating *H. pylori* in samples that were initially classified as negative by conventional methods. The researchers concluded that this was due to the small number of bacteria that conventional methods were unable to detect. It is worth noting that NGS not only allows for the detection of *H. pylori*, but also allows for the examination of a wide spectrum of basic mechanisms, such as the presence of genes associated with bacterial virulence, biofilm formation, restriction-modification systems or transcriptional response to exposure to various factors [20]. In turn, Min et al. used NGS to develop a panel for personalized *H. pylori* eradication treatment oriented to multiple species which targeted the regions for resistance to several antibiotics in *H. pylori* and human proton-pump inhibitor metabolism [58].

The use of NGS in the diagnosis of gastrointestinal infections seems justified when dealing with a patient showing clinical symptoms of infection but culture gives negative results. Then, it is possible to check not only for the possible presence of *H. pylori*, but also other bacteria that may cause similar symptoms. Such a diagnostic approach will save time because one reaction is enough to have insight into the entire stomach microbiome of the patient; based on the sequencing results, it is possible to assess the probable etiological factor of the infection, without the need to prepare several separate PCR reactions targeting different bacteria. This advantage of NGS is also described by Brown et al. [59] in the case of encephalitis of unknown etiology. Many cases of encephalitis remain undiagnosed because PCR and serology (which are commonly used) have limited and targeted detection. Given the myriad of rare pathogens that can cause encephalitis, this approach does not allow for the identification of new or unexpected pathogens that would be possible with NGS. The authors indicate that in as many as 28 cases out of 44 patients in which the etiological factor could not be diagnosed using PCR, NGS detected new (18/44), rare (5/44) or unexpected (5/44) microorganisms causing encephalitis [59]. This confirms the validity of using NGS in diagnostically difficult cases and allows us to assume that NGS would also find its application in difficult cases of gastritis.

In summary, the results of this study suggest that molecular methods, including NGS, can potentially augment the current approaches commonly used for the diagnosis of *H. pylori*. NGS is characterized by high sensitivity and specificity and, most importantly, provides repeatable and reliable results. On its own, NGS can serve as an alternative to PCR in *H. pylori* diagnosis, but not from an economic perspective. Due to its high cost, it may be worth considering when another infectious etiology is also suspected, as it enables comprehensive pathogen detection. Meanwhile, PCR-based methods, including “in-house” PCR-HRM, appear to be a good choice for routine diagnostics if an invasively collected sample is available.

Although molecular methods, including PCR, HRM and NGS, provide rapid and sensitive detection of *H. pylori*, bacterial culture from biopsy samples remains the gold standard for confirming infection and for performing antibiotic susceptibility testing. Such testing is critical for guiding effective eradication therapy, especially in the context of the global emergence of multidrug-resistant *H. pylori* strains, which are increasingly reported worldwide. MDR strains complicate treatment and require tailored antibiotic regimens based on susceptibility profiles [60,61]. Therefore, culture-based methods complement molecular diagnostics by enabling personalized treatment and monitoring of resistance trends.

## 5. Conclusions

The findings of this study suggest that molecular methods, including NGS, could enhance the current diagnostic approaches for *H. pylori*. NGS is distinguished by its high sensitivity and specificity, and, most importantly, it provides consistent and reliable results. Due to its high cost, it may be more worthwhile to consider NGS when another infectious cause is suspected, as it allows for the comprehensive detection of multiple pathogens. Meanwhile, PCR-based methods, including “in-house” PCR-HRM, appear to be a good choice for routine diagnostics of *H. pylori* infections in the pediatric patient group, if a biopsy specimen is available.

## Figures and Tables

**Table 1:** Cq values of the real-time PCR-positive samples and results of *ureA* gene-based PCR-HRM compared to NGS results (*n* = 16), all applied for detection of *Helicobacter pylori* DNA.

Sample No.	Real-Time PCR (Cq Value)	PCR-HRM for *ureA* Gene Presence	NGS Result (Non-Chimeric Read Outs Number)
1	+ (23.04)	+	+ (13,434)
2	+ (24.30)	+	+ (7768)
3	+ (22.52)	+	+ (24,498)
4	+ (19.47)	+	+ (42,925)
5	+ (22.45)	+	+ (7824)
9	+ (22.70)	+	+ (17,751)
27	+ (18.86)	+	+ (13,204)
29	+ (17.51)	+	+ (33,954)
31	+ (32.21)	+	−
32	+ (22.70)	+	+ (21,780)
40	+ (22.77)	+	+ (12,640)
41	+ (20.45)	+	+ (9145)
42	+ (20.47)	+	+ (18,912)
44	+ (20.87)	+	+ (8903)
45	+ (22.92)	+	−
48	+ (25.65)	+	+ (9619)

“+”—positive result, “−”—negative result.

**Table 2:** Comparison of the results obtained using real-time PCR and NGS.

Method	Result	
	Positive	Negative
real-time PCR	16 *	24
NGS	14	26

* all 16 samples were also positive in PCR-HRM.

## Data Availability

The raw data supporting the conclusions of this article will be made available by the authors on request.

## Supplementary Materials

The following supporting information can be downloaded at: https://www.mdpi.com/article/10.3390/microorganisms13102344/s1, Figure S1: Fluorescence curves for particular samples; investigation using “in house” High Resolution Melting PCR technique for *ureA* gene; Figure S2: Melting curves for particular samples; investigation using “in house” High Resolution Melting PCR technique, showing specificity for *ureA* gene; Figure S3:. Melting peaks for particular samples showing specificity for *ureA* gene amplicons; investigation using “in house” High Resolution Melting PCR technique.

## Abbreviations

The following abbreviations are used in this manuscript:

Cq	quantification cycle
NGS	next-generation sequencing
UTI	urinary tract infection
PCR	polymerase chain reaction
real-time PCR	real-time polymerase chain reaction
