# Supplementary material for: Comparison of Next-Generation Sequencing, Real-Time PCR and HRM-PCR for Helicobacter pylori Detection in Pediatric Biopsies
Source: Microorganisms. 2025 Oct 13;13(10):2344. doi: 10.3390/microorganisms13102344 (PMC12566025; doi:10.3390/microorganisms13102344)
Supplement: Supplementary file 1 [file microorganisms-13-02344-s001.zip › microorganisms-3877025-supplementary.pdf]

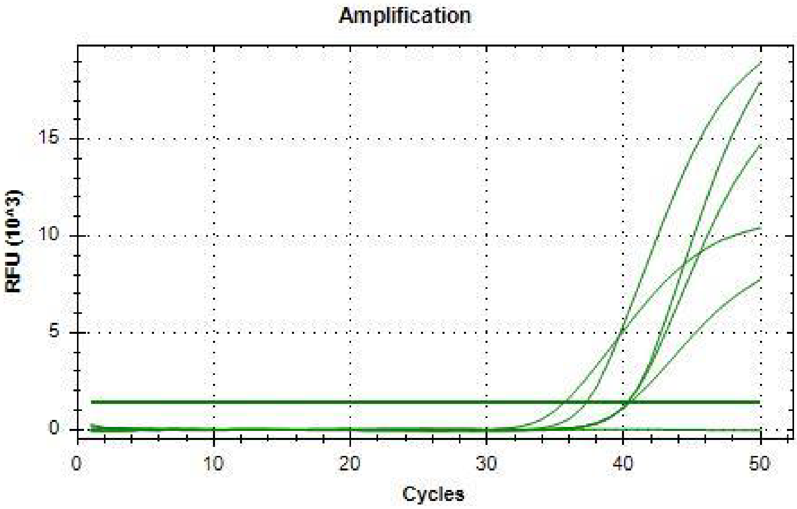

Figure S1. Fluorescence curves for particular samples; investigation using “in-house” high resolution melting PCR technique for *ureA* gene.

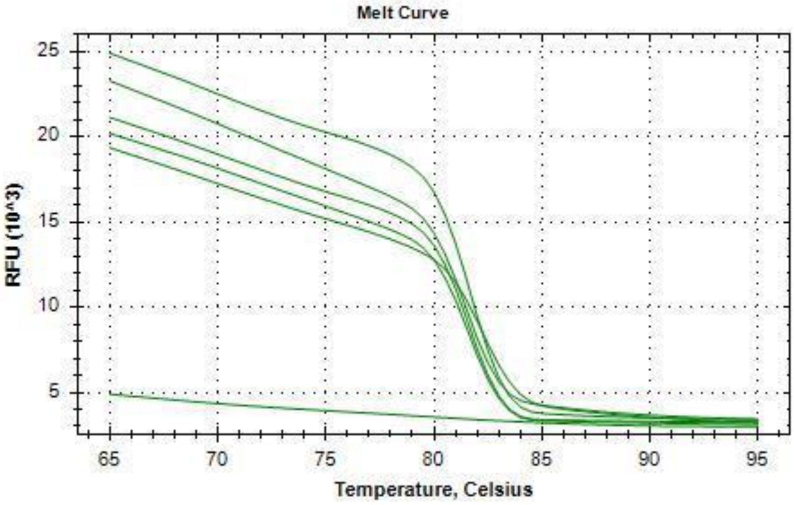

Figure S2. Melting curves for particular samples; investigation using “in-house” high resolution melting PCR technique, showing specificity for *ureA* gene.

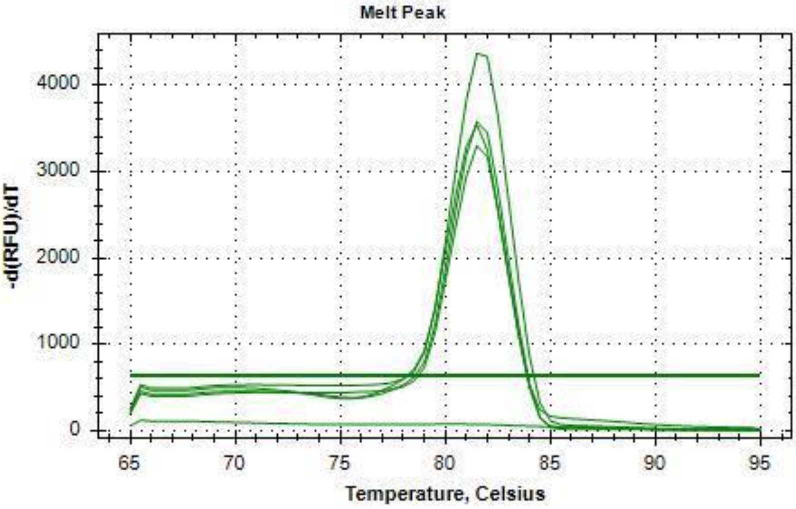

Figure S3. Melting peaks for particular samples showing specificity for *ureA* gene amplicons; investigation using “in-house” high resolution melting PCR technique.
